# Hyperdimensional computing: A fast, robust, and interpretable paradigm for biological data

**DOI:** 10.1371/journal.pcbi.1012426

**Published:** 2024-09-24

**Authors:** Michiel Stock, Wim Van Criekinge, Dimitri Boeckaerts, Steff Taelman, Maxime Van Haeverbeke, Pieter Dewulf, Bernard De Baets

**Affiliations:** 1 KERMIT Research Unit, Department of Data Analysis and Mathematical Modelling, Ghent University, Ghent, Belgium; 2 Biobix Research Unit, Department of Data Analysis and Mathematical Modelling, Ghent University, Ghent, Belgium; 3 Laboratory of Applied Biotechnology, Department of Biotechnology, Ghent University, Ghent, Belgium; 4 BioLizard nv, Ghent, Belgium; Indian Institute of Technology Mandi - Kamand Campus: Indian Institute of Technology Mandi, INDIA

## Abstract

Advances in bioinformatics are primarily due to new algorithms for processing diverse biological data sources. While sophisticated alignment algorithms have been pivotal in analyzing biological sequences, deep learning has substantially transformed bioinformatics, addressing sequence, structure, and functional analyses. However, these methods are incredibly data-hungry, compute-intensive, and hard to interpret. Hyperdimensional computing (HDC) has recently emerged as an exciting alternative. The key idea is that random vectors of high dimensionality can represent concepts such as sequence identity or phylogeny. These vectors can then be combined using simple operators for learning, reasoning, or querying by exploiting the peculiar properties of high-dimensional spaces. Our work reviews and explores HDC’s potential for bioinformatics, emphasizing its efficiency, interpretability, and adeptness in handling multimodal and structured data. HDC holds great potential for various omics data searching, biosignal analysis, and health applications.

## Introduction

Computational biologists and bioinformaticians collect, organize, process, and analyze large amounts of biological data to extract biological knowledge [1]. Parallel advances in biological data generation and computer science have further expanded the capabilities and usefulness of bioinformatics, proving immensely valuable for uncovering biological knowledge from sequence data. Today, bioinformatics is being transformed once again by deep learning (DL) [2] and its ability to handle complex, high-dimensional, and multimodal data such as sequences and images, redirecting interest from earlier powerhouses such as kernel-based learning [3,4]. Prominently, the development and use of complex DL models such as AlphaFold [5], ESMFold [6], and RoseTTAFold [7] represent a paradigm shift in protein structure prediction. Moreover, DL is also leading to significant breakthroughs in other fields, such as protein design [8], medical imaging [9], and drug discovery [10], with modest progress in fields such as systems biology and phylogenetic inference [11]. Many advances in deep learning for bioinformatics problems leverage novel neural architectures, such as the transformer [12], which was originally developed for natural language processing [13]. Curiously, there is a disparity between the fields in which the impact of DL can, to some extent, be explained by only the need for learning a mapping based on large data sets (e.g., protein structure prediction) versus the fields in which the problem settings involve complex combinations of structured data and information (e.g., multi-omics and phylogeny).

Two limitations currently hamper the utilization of DL models in bioinformatics [11]. Firstly, large connectionist models are often black boxes, while the explainability of models is an essential property for biologists, arguably more so than predictive performance. For example, when medical practitioners use a model to aid in making a diagnosis or finding a treatment, they must understand why this conclusion was reached [14]. Despite the great strides in explainable machine learning [15,16], DL methods still lack the clarity inherent in methods such as decision trees or logistic regression. Some authors argue that data-to-decision pipelines require truly interpretable models instead of explanations for black-box models [17]. Secondly, DL models are typically costly to train in terms of the required data and the associated computational cost. Most DL methods are very data hungry—with some notable exceptions, e.g., a recent RNA folding model trained on only 18 structures [18]. Training a single competitive DL model may cost tens to hundreds of thousands of US dollars and has a high environmental cost regarding CO_2_ emissions [19]. Meanwhile, transfer learning and fine-tuning have emerged as approaches to circumvent large additional training costs [20]. Furthermore, developing efficient architectures and training protocols is an active area of research [21,22].

This work explores the potential of hyperdimensional computing (HDC), sometimes called vector symbolic architectures (VSAs), as an alternative learning and information processing paradigm for bioinformatics [23]. While abstract models of the brain inspire HDC and DL, they are very different. Rather than mimicking the hierarchical connectionist neural architecture, HDC is a conceptual model of how representations are stored in the human brain. Here, concepts are represented by high-dimensional vectors (i.e., 10,000 dimensions or more), the eponymous hypervectors (HVs). HDC uses a set of mathematical operations to combine and change the information stored in different HVs to create an associative memory, a database of concepts. Using a small set of mathematical operations, one can construct, process, combine, split, or query the concepts in this database. For high-dimensional vectors, one can show that similarity metrics such as the cosine similarity or similarity based on the Hamming distance are extremely sensitive for detecting related vectors. Rather than being based on exact, algorithmic computing, HDC uses a cybernetic form of computation [24] where concepts are stored in a distributed fashion. Inferences are made by computing the similarity between query HVs and those stored in a memory, similar to how nearest-neighbor and other prototype methods work. Having many attractive characteristics that will be explored in the sections below, we believe HDC is a promising complementary paradigm to DL in bioinformatics with a wide range of applicability.

In the next section, we first outline the characteristic aspects of HDC. Then, we provide an accessible, though relatively comprehensive, introduction to HDC, including the different strategies of creating HVs, the basic operations and their intuition, how to represent the most commonly used data types (numbers, vectors, sequences, graphs, etc.), and how learning is performed. Finally, we discuss the strengths and promising applications of HDC for bioinformatics and computational biology. Though HDC is considered an obscure topic in some circles, there exists a vast amount of exciting work, which we cannot cover comprehensively. This paper should also serve as a general introduction for computational life scientists. We point to other work that is more specific or broader in scope.

## The nature of hyperdimensional computing

The HDC framework emerged in the nineties [25,26] and has recently seen a surge of interest in the machine learning community. It originates from a broader range of computational models that, by implementing an efficient binding operation to combine different sources of information, attempt to combine the benefits of so-called old-fashioned symbolic AI with the more modern connectionist and data-driven machine learning approaches. After the introduction of tensor product representations [27], many similar models, such as holographic reduced representations [28], binary spatter codes [29], and multiply-add-permute [30], have been suggested. Today, various models exist to construct HVs, for example, using binary, real-valued, or complex components. For an extensive overview, we refer to [26,31].

**Fig 1:**
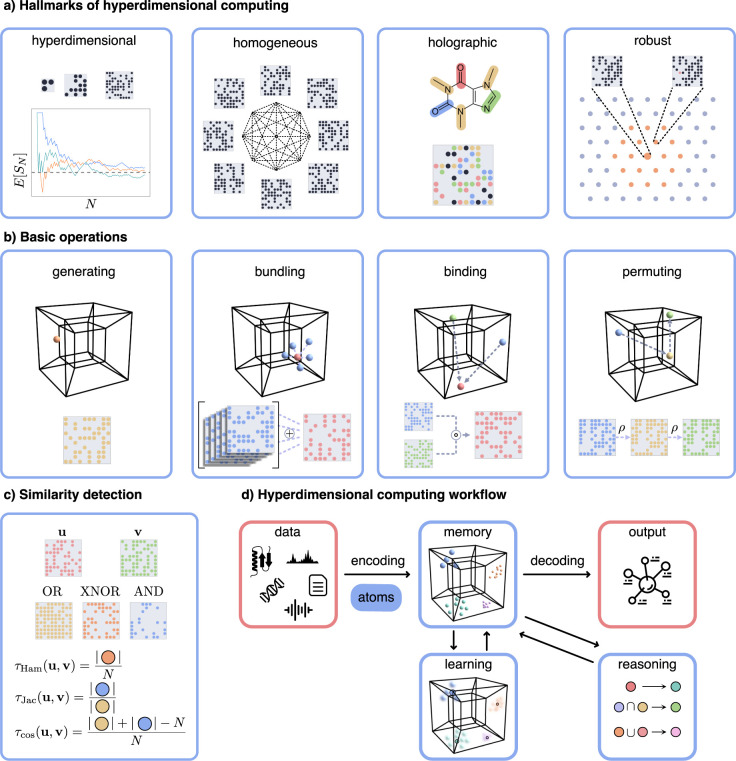
(a) The hallmarks of HDC. HVs work reliably due to their large dimensionality *N* (i.e., the Law of Large Numbers states that component-wise properties *S*_*N*_, such as the fraction of positive components, converge to their expected value for large *N*), and the space is very homogeneous (e.g., most HVs are approximately equidistant). The information about an object is encoded holographically, and the information is robust to random errors. (b) Overview of the elementary operations of HDC: generating, bundling, binding, and permuting. (c) Similarity is computed based on component-wise comparisons. (d) General HDC workflow, based on Thomas, Dasgupta, and Rosing [32], where red boxes indicate the data space and blue boxes indicate operations in the hyperdimensional space.

Rather than the specific choice of the values in the hyperdimensional representations, we identify 4 hallmarks that distinguish hyperdimensional computing from other approaches (Fig 1A). These are:

1. **hyperdimensional**: the HVs live in a very high-dimensional space, large enough such that random components can be seen as distinct and dissimilar from one another;
2. **homogeneous**: the vast majority of HVs all have highly similar properties: they have (approximately) the same norm, are all equally (dis)similar to one another, and have the same dimensionality, even if they embed more complex information, etc.;
3. **holographic**: the information encoded in an HV is distributed over its many dimensions; no specific region is more informative than another for a specific piece of information;
4. **robust**: randomly changing a modest number of the components does not substantially change an HV’s meaning.

These hallmarks characterize the nature of the HVs, and together with a well-chosen set of mathematical operations, they allow one to encode complex structures such as amino acids, genes, gene regulatory networks, proteins, or whole genomes. As a general guide, information is preserved using *similarity*: similar entities or complex constructions have similar HV representations. A sensitive similarity measurement, such as one based on the Hamming distance or the Jaccard similarity, is vital for inference and querying.

The first vital aspect of the power of HDC is the **high dimensionality**, typically around 10,000 as a guideline. It leads to an astronomical representational power for complex objects such as genes or networks: 2 randomly selected vectors will likely be dissimilar, allowing them to store information independently. Hyperdimensionality also leads to robust systems, a phenomenon known in mathematics, statistics, and physics as *the blessing of high-dimensionality*. For example, in statistical physics, systems with many degrees of freedom lead to robust behavior that can be described by emergent properties such as pressure [33]. Different data types are encoded in the same form of HVs, a property we call **homogeneity**. One cannot tell whether a specific HV encodes a more complex concept, such as a protein, or an atomic concept, such as an amino acid. In HDC, individual components of the HVs cannot be linked to specific information about an entity. Instead, all components contribute slightly to representing all the properties at once. This distributed representational property is called **holographic**. Storing a little bit of all the properties in every component is the basis for homogeneously constructing complex objects. This property is in marked contrast with, for example, TF-IDF word embedding vectors (each component corresponds to the occurrence of a word in a particular text) or molecular fingerprints (components correspond to the occurrence of specific subgroups of the molecule). Finally, HDC is **robust to noise** because of the above properties. Due to its holographic nature, each component comprises the same information but in a differently corrupted way, such that hyperdimensionality ensures a representation that is inherently robust to corruption. This will allow for the construction and manipulation of complex entities without too much loss of information. In essence, computing similarities of large, randomly initialized vectors can be seen as approximating expected values, which are preserved under unbiased corruption, i.e., noise.

## A gentle introduction to HDC

### Computing with HVs

The basic operations needed for HDC are remarkably simple. In brief, they hinge on 4 operations to manipulate and extract the information in the HVs (Fig 1B and 1C). These are the following:

- generating new HVs from scratch;
- combining a set of HVs into a new HV that is similar to all;
- using one or more HVs to generate a new one that is dissimilar to its parent(s);
- comparing 2 HVs to detect whether they are more (dis)similar than expected by chance.

This work will limit the discussion to the most basic, generally used cases. For an exhaustive overview, we refer to survey papers such as [26,34]. We also refer to [31], who compare eleven different HDC architectures in-depth and [32] for theoretical analysis. As a running example, we will use the encoding of amino acid sequences to explain the operations.

#### Hypervector generation

Firstly, one has to fix the nature of the HVs, e.g., whether to work with binary ({0, 1}), bipolar ({–1, 1}), ternary ({–1, 0, 1}), sparse, or real-valued vectors. One needs to define a function for these types to generate new *atomic vectors*. Atomic vectors represent the basic building blocks of the entity of interest. For example, protein sequences consist of amino acids, DNA sequences of nucleotides, and protein networks of proteins. These are atomic in the sense that they are, within their context, not composed of simpler substructures. In practice, generation can be done by initializing an *N*-dimensional vector with i.i.d. (pseudo-)random numbers of the appropriate type, e.g., Booleans drawn from a Bernoulli distribution, −1 and 1 drawn from a Rademacher distribution, or with normally distributed values. The high dimensionality ensures that these randomly generated vectors satisfy the properties described earlier.

#### Bundling

Given a collection of HVs, *bundling* (also called aggregation or superposition) yields a HV similar to all elements in the collection. For example, bundling 3 HVs is denoted as follows:

$$u=[v_{1}+v_{2}+v_{3}],$$

where […] denotes a potential normalization operation. In the case of binary HVs, for example, normalization corresponds to thresholding such that **u** is again a binary HV, and aggregation boils down to a component-wise majority rule. Here, we have that **u** ~ **v**_1_, **u** ~ **v**_2_ and **u** ~ **v**_3_ where “~” informally denotes that 2 HVs are similar, i.e., they are related. In the case of binary (0/1) or bipolar (−1/1) HVs, “similar” means that they share more components than expected by chance.

As an example, consider the task of finding a HV that represents the set of all hydrophobic amino acids. For binary HVs, one could solve the closest string problem, an NP-hard problem that finds the bitstring with the smallest Hamming distance to all the given HVs. In practice, however, one often uses a much simpler method: the HVs of the hydrophobic amino acids are bundled using component-wise majority. When bundling an even number of components, one has to adopt a convention to resolve ties by setting a default value or randomly picking one. Bipolar HVs are particularly easy to bundle, as one can add the vectors and take the sign of the components; in the case of ties, one can use 0 as a neutral component and upgrade to ternary HVs. Taking the average for real-valued HVs seems appropriate, though this will reduce the aggregate’s norm, violating the homogeneity property. This decrease in norm can easily be understood from the variance rule for independent random variables:

$$Var\left[\frac{X_{1}+X_{2}}{2}\right]=\frac{Var[X_{1}]+Var[X_{2}]}{4}.$$

To bundle *n* HVs, it is better to either compute *n*^−1/2^ ∑_*i*_ **v**_*i*_ or to renormalize the sum to match the norm of an atomic HV.

#### Binding

Though powerful, bundling alone cannot represent complex, hierarchical structures. For example, suppose one has the dimer AC (alanine and cysteine) and the dimer CE (cysteine and glutamic acid). In that case, one cannot directly create a bundling from which the identity of these dimers can be recovered. A superposition of both dimers would represent a bag of amino acids, unable to specify which nucleotides are connected to each other in a dimer. This problem is called the *superposition catastrophe* [35]. Binding, denoted by ∘, solves this problem by generating a new vector from 2 old ones:

$$u=v_{1}\circ v_{2},$$

such that **u**≁**v**_1_ and **u**≁**v**_2_, where “≁” indicates that the vectors are not similar. For bit vectors, component-wise, XOR-ing serves well. For bipolar or real-valued HVs, one typically uses component-wise multiplication, though alternative binding operations such as circular convolution [28] are also used. Importantly, binding is often reversible and does not destroy the information, i.e., there is an *unbinding* operator ⊘ that reverses the binding and releases the bound information:

$$v_{1}⊘u=v_{1}⊘(v_{1}\circ v_{2})=v_{2}.$$

For binary and bipolar HVs, binding and unbinding are the same operations, e.g., XOR is self-inverse. Combining bundling and binding allows one to store a data record, i.e., a set of key-value pairs **u**_1_∘**v**_1_,…,**u**_*n*_∘**v**_*n*_, which one can query as follows:

$$u_{i}⊘[u_{1}\circ v_{1}+\cdots +u_{n}\circ v_{n}]=[v_{i}+noise]\approx v_{i}.$$

The above operation is one of the central ideas behind inference with HVs. For example, by storing a collection of sequences with a bound functional annotation (e.g., enzymes with their associated EC numbers), one can query with new sequences to obtain the likely function annotation. Additionally, this data record encoding is a generic template for encoding different types of data, allowing to store feature identifiers as keys and their associated values.

#### Permutation and shifting

A special case of binding is binding by *permutation*, creating a variant *ρ*(**v**) of a single HV **v** such that

ρ(v)≁v.

Permutation generates a concept variant, such as the phosphorylation of a protein or the methylation of a nucleotide. The permutation is often implemented as a circular vector shifting with one or more positions, denoted as *ρ*^*i*^(**v**). Typically, one can easily invert this operation by shifting the corresponding number of positions in the opposite order, i.e., *ρ*^−*i*^(**v**). Permutation is often used to generate bindings of sequences that retain order information. For example, one can embed the amino acid sequence GNP as

$$\rho^{1}(v_{G})\circ \rho^{2}(v_{N})\circ \rho^{3}(v_{P})$$

from the respective amino acid HVs.

#### Similarity

The above operations suffice to create arbitrarily complex structures in the hyperdimensional space. One can extract information from this space by comparing HVs based on *(dis)similarity*. A meaningful similarity measurement is vital for performing *inference*. One often tries to find the entity in the data space that matches the HV result most closely, either by search or optimization. Typically, the large dimensionality ensures that the similarity between 2 arbitrary HVs is tightly bound, leading to an extremely high sensitivity to detect related HDs.

For bit vectors, one often uses similarities based on the Hamming similarity. The normalized Hamming similarity is given by

$$\tau_{\mathrm{Ham}}(u,v)=\frac{1}{N}\sum_{i=1}^{N}\delta_{u_{i}},v_{i},$$

with *δ*_*x*,*y*_ the Kronecker delta function, yielding 1 if *x* = *y* and 0 elsewise. This relative Hamming similarity yields values between 0 and 1, with 2 randomly generated vectors having a value of 0.5.

In bioinformatics, the Jaccard index (often called the Tanimoto similarity in the comparison of chemometric fingerprints [36]) is a popular alternative. It is the ratio of the number of components that equal 1 in both vectors to the number of components that equal 1 in at least one of the vectors:

$$\tau_{Jac}(u,v)=\frac{u\cdot v}{u\cdot u+v\cdot v-u\cdot v}.$$ (1)

The Jaccard index also yields values in [0,1] with 1/3 the expected value for comparing 2 random vectors (0.5^2^/(1–0.5^2^)). Since the Jaccard index is appropriate for comparing sets, every position of the HVs is interpreted as a holographic property that the entity does or does not possess, similar to how molecular fingerprints yield information on whether a subgroup is present or absent in a molecule.

For bipolar or real-valued HVs, the cosine similarity is a more natural choice:

$$\tau_{cos}(u,v)=\frac{u\cdot v}{\left|u||v\right|}.$$

Here, the output ranges from −1 to 1, and the similarity of 2 randomly generated vectors is expected to be close to 0.

## Encoding of data types

Armed with the 4 basic operations of HDC, one can map all kinds of objects, such as sequences, graphs, or vectors, into the hyperdimensional space. Several strategies exist for all the different data types. As often in data science, some feature engineering might be required to obtain the best representations for a given application. As a general guideline, similar objects should result in HVs with an increased similarity. We refer to [26] for a more comprehensive survey.

### The atomic building blocks

The first step for a given data type is typically identifying the atomic building blocks (e.g., amino acids for protein sequences or proteins for protein–protein networks) and representing them using random generation. Next, these can be combined into structured hierarchical object representations using bundling, binding, and permutation.

***Symbols*.** Atomic building blocks, such as symbols representing a unique concept, can be generated directly. These symbols might, for example, represent the characters of biological sequences, metabolites, or elements from some ontology. Because of the hyperdimensionality, randomly generated HVs are all dissimilar, meaning that these concepts can be seen as independent. If one wants to encode that one concept is semantically closer to another concept, one can randomly copy a small fraction of one HV to the other, making them more similar [37]. A more general way of embedding semantic information in HVs is embedding them into a graph where semantically similar concepts are connected and optimizing the HVs to minimize an energy function over this graph [38,39].

***Scalars*.** Like nodes in a graph, scalars are another data type where some values are semantically closer to one another than unrelated symbols. For scalars, it is vital to incorporate the notion of closeness. Care has to be taken when the HV components are low-resolution, such as binary, bipolar, or ternary HV. A scalar, representing, for example, gene expression, is usually represented by considering a fixed range of values divided into discrete bins, with intermediate values obtained by interpolation. The HV representing one bin is typically constructed by randomly changing a fraction of components of the HV of the previous bin. This type of encoding originates from the scatter code [40]. One can achieve different similarity patterns with varying properties and resolutions by defining the bin width and the number of randomly changed components. The bundling of neighboring bin representations can be interpreted as an approximation of values right in between—alternatively, more continuous approaches without discrete bins exist [41,42]. We refer to [26] for a more detailed overview. Scalar encodings can also form the basis for regression using HDC.

### Composite objects

***Numerical objects*.** Numerical composite objects, such as real-valued vectors (e.g., gene expression vectors) or functions (e.g., dose-response curves), can be constructed using the above-mentioned atomic scalar representations and operations. For example, small vectors can be encoded by binding their scalar components, likely by shifting to encode the position. Alternatively, to encode a larger vector **x**, one can use a random projection

v=Sx (2)

where *S* is a random, potentially sparse projection matrix containing normally distributed values or components from {−1,1}. Some schemes, such as [43]’s BRIC, suggest a specific structure in the projection matrix to promote hardware optimizations. The resulting HV **v** might need to be thresholded, sparsified, or normalized [43]. Such random projections are well established with an extensive body of theoretical justification for why they retain the properties of **v**, e.g., the Johnson–Lindenstrauss lemma [44–46] or its sparser variants [47]. Additionally, using well-chosen numerical value encodings, more complex numerical objects such as functions and distributions can be approximated arbitrarily close using integral transformations [42,48–50].

***Sets and sequences*.** Sets can be represented as an aggregation in terms of their symbols. This aggregation acts similarly to a Bloom filter [32,51], a stochastic data structure used for checking whether an element is part of a set using multiple hash functions.

Sequences, such as DNA, RNA, or peptides, differ from sets in that the order of the symbols matters. Merely bundling the symbols would not suffice. To account for the order, one can encode the position using shifting, e.g., no shift for the first symbol in the sequence, a shift of one for the second symbol, and so on. One can form the HV of the sequence either by bundling, e.g.,

$$u=[\rho^{0}(v_{1})+\rho^{1}(v_{2})+\rho^{2}(v_{3})+\rho^{3}(v_{4})]$$ (3)

or using binding:

$$u\prime =\rho^{0}(v_{1})\circ \rho^{1}(v_{2})\circ \rho^{2}(v_{3})\circ \rho^{3}(v_{4}).$$ (4)

When using bundling, one can measure the similarity between 2 sequences based on their representation. An HV obtained by binding the sequence is dissimilar to the representation in which a single symbol differs. When encoding longer sequences, such as proteins or whole genomes, one typically uses the *n*-gram approach (often called *k*-mer in bioinformatics), using both binding and bundling. Here, one typically represents all subsequences of length *n* using binding, after which the *n*-gram representations are bundled into one sequence representation [52]. It might be beneficial to combine several different representations at different levels. For example, to encode a bacterial genome, one might combine multiple *n*-gram representations with a representation based on the presence of the different genes, themselves encoded based on their DNA and protein-coding sequences.

***Graphs*.** Graphs, such as metabolic networks, protein–protein networks, or molecules, are also structured datatypes consisting of vertices and edges. Vertices can be atomic or composite. Representations of an edge can be constructed by combining the representations of the corresponding nodes as done in GraphHD [53] and GrapHD [54]. One can directly bind the 2 node vectors if the graph is undirected. When the graph is directed, e.g., in gene-regulatory networks, one can shift one of the node vectors to distinguish between an ingoing and an outgoing edge. When all edges are encoded, they can be bundled to create an HV representing the entire graph. These HV representations allow for solving graph problems such as graph matching, shortest path finding, graph classification, and object detection. Specific methods such as Holographic Embeddings can even scale efficiently to very large data sets [55].

***Images*.** Images are the last data type we consider. An image is usually a 2D matrix in which the components represent the pixel values, either as brightness, color, or something more specialized, such as different channels of microscopy images. Again, one can represent the whole image by bundling the pixels with the appropriate spatial context. A simple way to encode this context is by defining 2 permutation types, representing the pixels’ coordinates [39]. This approach has the drawback of not accounting for the closeness between pixels. Representations based on role-filler binding [56] mediate this problem. Here, close positions are made more similar, in a similar way as we discussed for scalars. In [57], black-and-white MNIST images were processed directly in flattened format, in which each pixel location was represented by an atomic vector that was shifted or not, depending on the pixel value. This naive approach performed quite poorly compared to a CNN, with a reported training accuracy of only 86% compared to the 99% accuracy of LeNet. One effective alternative to creating image representations directly from the pixel values is using a hidden layer of a (convolutional) deep neural network (see, e.g., [58]). This strategy can also be used for other data types with associated pretrained deep neural architectures, such as (graph) convolutional neural networks or transformers.

### Learning with hypervectors

Here, we give an example of the practical learning flow for machine learning with HDC, depicted in Fig 1D. Hyperdimensional computing bears more than a passing similarity to kernel-based learning [3]. Both project the data to a higher-dimensional space to make the data patterns more easy to capture. However, whereas kernels typically use implicit mapping and linear algorithms in this space, HDC creates this space explicitly and mainly uses prototype-based learning. The majority of the work within HDC focuses on classification [26,59], but some variants for regression exist [41,60]. Typically, one first maps the data to HVs using the methods described in this section. Then, these encoded data points are processed for learning and reasoning using the operations in the hyperdimensional space. Finally, similarity measurements allow for mapping the processed HVs back to interpretable predictions.

More concretely, a classification, such as embedding variants of a particular protein with a function, is typically performed using *prototype methods* [59]. Each class has a prototype HV designed so that the classification of a new data point can be performed based on similarity measurement. The predicted class is the one for which the prototype HV is most similar to the HV representation of the data point to predict. Most HDC learning schemes can be seen as a specific instance of vector quantization [61] or its supervised variants [62,63].

Different heuristic algorithms exist to compute class prototype HVs. The basis is bundling all the HV representations of the members of a class. Although simple bundling is computationally efficient, easy to implement and often works reasonably well, it frequently falls short in predictive performance compared to other contemporary machine learning methods. The predictive performance can be greatly improved by various *retraining* algorithms. Typically, one cycles through the training set several times, during which wrongly classified examples are added to the correct class prototype and subtracted from the wrongly associated prototype [59,64]. For example, assume 2 classes *A* and *B* with initial hypervectors **C**_*A*_ and **C**_*B*_ obtained by bundling. If a data point, represented by hypervector **v**, is misclassified as *A*, then one updates **C**_*A*_ ← **C**_*A*_− *α***v** and **C**_*B*_ ← **C**_*B*_ + *α***v** with *α* the learning rate. Different variants with, e.g., data-dependent or iteration-dependent learning rates, exist to increase performance or speed of convergence [65].

## Strengths of HDC for bioinformatics

**Fig 2:**
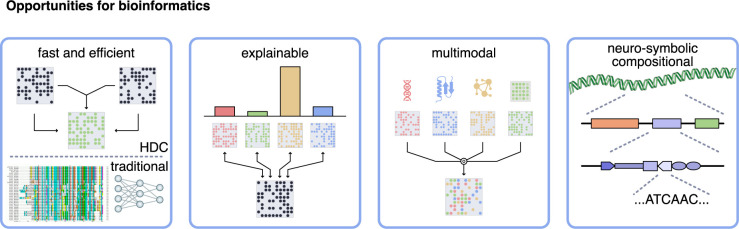
Opportunities for bioinformatics. (i) HDC is computationally efficient because it can usually be done using simple bit or arithmetic operations; (ii) it is explainable because of its reversibility; (iii) it can easily combine different types of data sources; and (iv) it can represent complex, structured, and hierarchical information.

Though HDC is gaining some prominence, it remains relatively underexplored for bioinformatics applications compared to other machine learning approaches. The HDC paradigm can be instrumental in bioinformatics because the field increasingly deals with large amounts of sequence data linked with knowledge. More specifically, in this work, we identify 4 opportunities that HDC can bring to the field of bioinformatics (Fig 2):

1. **fast and efficient**: HDC has the potential to be much faster than classical alignment algorithms or DL approaches;
2. **explainable**: the operations in HDC are tractable and often reversible, making them close to white box operations;
3. **multimodal**: all data are mapped to the same *N*-dimensional vectors, combining different sources of data (e.g., transcription and metabolomics or sequence and structure) is trivial;
4. **symbolic and hierarchical**: HDC is equipped with an algebra to reason about structured data, such as representing a gene construct.

A similar set of strengths was explored by [66] in the context of biosignal processing.

The field of bioinformatics is generating ever-growing amounts of data [67]. This is primarily due to plummeting sequencing costs, making reading expression possible from the individual cell level to the level of entire microbial communities. In addition, other rich and diverse data sources, such as metabolomics [68], imaging [69], and flow cytometry [70], are also becoming available in a high-throughput fashion. These data types require specific data processing algorithms to be analyzed and compared. For example, sequence analysis is driven by advancements in sequence alignment algorithms. Machine learning methods, and specifically DL architectures, represent flexible, trainable operations with unprecedented power—and often exorbitant computational demands! Hyperdimensional computing is seen as a time- and energy-efficient form of machine learning because processing is extremely fast despite the large size of the HVs, with speed improvements from 5 to 50 times compared to traditional methods reported [71]. For example, the review by [71] reports speedups ranging from a factor 2 to a factor 50 for various applications, often with a minor performance cost. The reason is that encoding, training, and inference usually require only simple component-wise operations. The operations can often be done using bit vectors, allowing for efficient low-level encoding. Due to the simplicity of the operations, HDC systems can be implemented on specialized hardware, such as GPUs [72,73], FPGAs [74,75], and memristors [76]. For example, Demeter [77], an HDC-based metagenomics profiler, used extensive hardware optimizations to attain more than a hundred-fold speed improvement and 30-fold memory improvement compared to Kraken2 [78] and MetaCache [79], while maintaining comparable accuracy.

There is a large gap between the vast amount of data and the generation of biological knowledge. Ideally, a model must be explainable to create robust predictions so the user can verify its assumptions, i.e., why something is predicted and not something else [14]. Many approaches exist toward explainable machine learning [80], either as models that are naturally interpretable or using post hoc analysis such as Shapley value analysis [81]. Symbolic regression methods can directly distill parsimonious, human-readable rules from data, often with great accuracy [82,83]. Given that HDC works with large, randomly constructed high-dimensional vectors, it is surprising that it is quite explainable. This explainability is due to HDC’s reversible operations, meaning one can decompose complex representations to learn how they work. One can use similarity matching to compare the HV with different components to see what is essential, for example, to learn which groups or combinations of groups are responsible for the biological activity of a molecule.

Bioinformaticians not only have to deal with more data, but these data are also becoming more diverse. Data fusion combines data from different modalities that provide separate and complementary views on common phenomena to solve an inference problem. Considering the different data sources to discover molecular mechanisms, sample clustering, or attaining the best predictions is far from trivial [84]. In precision medicine, for example, one can describe a patient’s health status using various omics, metabolites and biomarkers, the microbiome, wearable reading, and the environment [85]. Deep learning has shown considerable success in data fusion, as the hierarchical representation makes such models very suitable for multimodal learning [85]. In HDC, different data sources are mapped to the same vector types, bringing them to equal footing. Simple binding or more complex strategies can combine the different HVs into a single HV representing the different modalities of the object. For example, a fusion of different types of wearable sensor reading—electroencephalography recordings, accelerometers, galvanic skin response—can accurately detect human activity and emotions [86,87].

A final aspect where HDC shines is representing complex, structured hierarchical information. Biological data is inherently hierarchical and nested: protein networks consist of proteins, which include domains and amino acids. The most potent representations also incorporate the aspects of the lower-level constituents. However, combining complex information is a challenging problem, referred to as the binding problem [88]. For example, combining the concepts of a “red apple” and a “green pear” might lose the specific color-object associations. In bioinformatics, an example would be adding semantic information to individual genes in a set. The operations of HDC are well suited to handle this issue, allowing one to freely combine specific concepts due to the distributive properties of the operations. For example, in image processing, one can use bundling to combine several holistic image descriptors with local image descriptors of specific regions in the image for more accurate place recognition for mobile robotics [89]. This allows HDC to thrive for problems with reasoning and structure, such as recently outstripping DL on Raven’s progressive matrix problem [90].

Many tricky machine learning problems can become trivial using HDC. For example, suppose one has computed several energetically favorable RNA secondary structures, but one does not exactly know which one(s) is or are biologically active—an instance of multi-instance learning [91]. Such problems are ubiquitous in bioinformatics. They are easily handled by aggregating to obtain an HV similar to all candidates.

## Opportunities for bioinformatics

Here, we identify several domains in bioinformatics in which HDC has proved valuable. In addition, we also speculate on which other domains in bioinformatics remain to be explored and which domains might not be a perfect fit for HDC.

### Analyzing omics data at scale

HDC has proved its worth in processing omics data. Within the omics domain, problems usually involve matching high-throughput generated data to a reference database. This application is especially useful for HDC, given its speed and low memory footprint. Notably, because HDC works with fixed-shape representations, (sub)sequence matching becomes independent of the length of the reference sequence. Several studies have reported magnitudes of improvements in both speed and energy use compared to the state-of-the-art. HDNA [92], GenieHD [93], BioHD [94], and HDGIM [95] are HDC-based frameworks to match DNA sequences to reference databases efficiently. Often, these make use of highly parallelized implementations and specific hardware optimizations. For example, BioHD uses processing in memory (PIM) for massive parallelism to obtain 100× speedups and energy efficiency, even compared to other algorithms running on PIM accelerators. As a tool for protein back-translation, they resolve ambiguities in similar-encoding nucleic acid sequences by superposing them. Another example is Demeter [77], a metagenomics profiler made for real-time monitoring of food. The authors use specific memristor optimizations to obtain large memory reductions and speed improvements compared to state-of-the-art methods while seeing only negligible drops in accuracy. In epigenetics, HDC was successfully used to classify tumor and non-tumor sequences based on their methylation profile [96]. Alternative sequence encodings were proposed, improving performance on tasks such as protein secondary structure prediction [97]. These encodings provided equivariance concerning the shift of sequences and preserved the similarity of sequences with identical elements.

### Biosignals and spectra

A second domain with large amounts of data relates to biosignals and spectra. Here, HDC can also provide fast ways to analyze large-scale data at competitive performance. For example, HyperSpec [98] is an HDC-based approach for clustering mass spectrometry data that achieves speedups of up to 15-fold compared to alternative clustering tools. In addition, HyperSpec combines both the spatial locality of the spectra peaks and the intensity of those peaks, making it an excellent example of HDC’s advantage in coping with complex data. HDC has been used for classifying the sensitivity of glioma to chemotherapy using proteomics SELDI-TOF spectra [99]. Similarly, the recently proposed HyperOMS [100] is an HDC-based algorithm for open modification spectral searching in mass spectrometry proteomics for identifying posttranslational modifications.

Related to biosignal processing, a prominent example using HDC is the work by [101], who developed a set of HD architectures for encoding ExG signals across multiple modalities and demonstrated how these architectures result in explainable HVs. In later work, [66] extensively explored the potential of HDC for various ExG biosignals (i.e., electromyography, electroencephalography, and electrocardiography) and found equal to superior performances compared to the state-of-the-art, while HDC (i) demanded much less data and could work in the zero-shot setting; (ii) dealt well with noisy and unprocessed inputs; and (iii) proved to be transparent and repeatable. In general, a lot of work within HDC has focused on the processing of bio(medical)signals, often on IEEG, EEG, or EMG signals, for example, in seizure detection, septic shock modeling, and hand gesture recognition [66,102–125].

### Molecules and graphs

HDC methods are also suitable for learning with molecules and graphs. Graphs and networks are invaluable tools in systems biology, for example, in metabolic networks, protein–protein networks or gene regulatory networks. GraphHD [54] and GrapHD [53] are general HDC-based approaches for encoding and classifying graphs, achieving comparable performance as state-of-the-art methods on real-world classification problems. HyperRec [126] is a recommender system based on HDC. The method encodes items and users into HVs to predict rankings for new items based on user preferences, which can be seen as predicting links in a graph. Graphs containing drug–drug, protein–protein, and drug–target interactions were represented as HVs to predict new adverse drug–drug effects [127]. Also, the hierarchical structure of atoms in a molecule represents a graph and can used to predict molecular properties based on HDC [128]. MoleHD [129] is a more recently proposed HDC tool for molecular property prediction, such as permeability through the blood–brain barrier or drug side effects. This method performs favorably compared to several state-of-the-art methods, including graph convolutional neural networks, while requiring mere minutes to train on a CPU, as opposed to days to weeks of GPU training times for the deep-learning-based approaches.

### Online and precision healthcare

HDC is also being applied in the domain of online health care. It is an excellent fit due to its efficiency and multimodal-friendly characteristics. For example, [86] developed HDC-MER, an HDC framework for emotion recognition based on multiple modalities that are encoded into HVs across time. [102] also developed an HDC method for emotion recognition but specifically leveraged a cellular automaton for HV generation to maximize energy efficiency in settings with many modalities. A third example is the work by [130], in which a proposed HDC method can analyze computed tomography scans for early COVID-19 detection. Furthermore, HDC has been applied for seizure [104] and septic shock detection [110], in overlap with the works cited earlier on the processing of biomedical signals.

### Text mining

HDC was used for natural language processing and analogical retrieval of information using predication-based semantic indexing [131,132]. For example, from the composition in the sentences such as “drug A *treats* disease B,” one may infer the predicate pathway “drug A *interacts with* gene C *associated with disease B*.*”* In this way, HDC can be used to mediate the identification of therapeutically valuable connections for literature-based discovery [133]. Similarly, HDC was discussed in the context of pharmacovigilance, drug repurposing, and discovery-by-analogy [134,135]. Note that these (analogical) interferences of interactions based on language show a strong connection to the more explicit graph representations discussed earlier, emphasizing the multi-modality of HDC. The scale at which modern DL techniques can process large, diverse data sets has given rise to *foundation models*, general models capable of being adapted to a wide range of downstream tasks [136]. Large models, such as BioBERT [137], allow for the processing of large amounts of biomedical data, for example, for drug discovery or personalized medicine [138]. HDC can complement such approaches as its strengths complement the weaknesses of DL, i.e., HDC systems being lightweight to train and deploy and their transparent operations. Here, HDC is suited to create relatively small, highly specialized knowledge systems.

Finally, HDC is also used in medical imaging [139]. For example, [140] used fMRI images for biological gender classification, and [130] used CT scans for detecting COVID-19-related pneumonia.

### Other opportunities

Beyond areas where HDC is already being applied, we also see opportunities to apply HDC in yet-to-be-explored areas. Phylogeny is the first domain where HDC could shine. In the field of high-throughput genomics, there is a strong need for novel and performant phylogenetic methods, for example, to link genomic features and traits based on macroevolutionary genomic data [141]. HDC would be a powerful and versatile alignment-free method [142], which can be more flexible and computationally performant than traditional alignment-based methods (though the former might be less accurate), especially when sequences are not homologous. There are many approaches to alignment-free methods, but there is no clear general best [143]. Many of them are based on *k*-mer counts or numerical representations, similar to how HV embeddings are constructed. For example, Li and colleagues [144] found that numerical vectors of nucleotide composition could lead to excellent phylogenetic trees throughout the Tree of Life. Hypervectors can incorporate both the location information and physicochemical information of the *k*-mers. They allow for incorporating various data sources—genomics, expression, morphology—in simple vectors that can be compared directly to build a tree. The hierarchical nature of HDC would allow one, for example, to encode all the gene variants of a species and combine these in an HV that represents their relative order in the genome. This would, for example, allow for studying organisms with complex mosaic genomes, such as phages [145].

A final application in which we see a lot of potential for HDC is genetic engineering, biotechnology, and breeding. Such endeavors are often very specialized projects, frequently of a proprietary nature, in which a lot of domain knowledge and experimental data are available. For example, enzyme engineering combines wild-type sequence data in their biological context with various mutation experiments and activity and stability assays. This information has to be integrated into a model that correctly incorporates the causal mechanisms so that the most promising new mutations can be highlighted. Synthetic biology is modular in the sense that the basic parts, genes, protein domains, or cells can be combined into new functional entities [146]. The composability of HDC can be suitable to represent such designs.

## Limitations of HDC

Some application areas are likely less relevant for HDC. These are areas where highly complex relationships need to be learned with rather limited knowledge and where one can rely on adequate objective functions for optimizing a complex black-box function: cases where DL shines. One example is protein structure prediction, in which the goal does not match the strengths that HDC can offer. A second area is generative applications such as protein design. Although HDC methods can be generative, we believe that the goal of protein design needs to be aligned better with the strengths of HDC frameworks. In general, DL’s strength is in learning to map from one space to another, given that these spaces are densely populated with examples. HDC, however, shines when there is a specific, known structure that one wishes to encode.

In general, obtaining a performance that is as good as that of conventional machine learning algorithms can be tricky for some applications. For example, in [71], it is observed that while HDC can perform state-of-the-art for 1D data, such as text, sound and biosignal classification, its performance on 2D data, such as images, is still inferior. The large dimensionality of the HVs also incurs a large memory footprint, for which clever implementation or hardware accelerations are needed to attain high speeds. Like kernel-based methods, the flexibility of representing data in HDC also has the drawback that substantial feature engine ering might be required for good encoding. To attain competitive predictive performance, one usually needs some retraining scheme.

## Conclusions

A key idea in bioinformatics is that statistically meaningful similarities indicate a biological signal, a reasoning often based on evolutionary principles. Many alignment-based algorithms for sequences, structures, or graphs exploit this principle by searching large databases for homologs and the like. More recent approaches based on machine learning, specifically deep learning-based approaches, have been highly successful at learning general maps from complex input to output domains, such as sequence to structure [5]. Their power and generality have transformed nearly every subdomain of bioinformatics.

This work discussed hyperdimensional computing as an additional tool in the bioinformatician’s arsenal. Hyperdimensional computing shares similarities with search-based and learning-based paradigms, such as kernel methods. Hyperdimensional computing’s strengths nicely complement some of the weaknesses of deep learning (and likely *vice versa*). Initially, the most prominent selling point of hyperdimensional computing appears to be its speed and computational efficiency, allowing for training on parallel hardware and performing online inferences at scale on specialized hardware such as FPGAs [147]. Extensive benchmark studies for different applications will be critical for making the right design choices.

Future “unconventional computation” strategies might use alternative physical, chemical, or biological processes for computation, such as optics [148], reaction-diffusion processes [149], or plants [150]. Hyperdimensional computing would be well suited for such forms of stochastic cybernetic modes of computation [24].

The ability of hyperdimensional computing for structural compatibility using algebraic operators might be even more useful than its computational efficiency. These operations allow the bioinformatician to encode prior domain knowledge and the problem structure in the predictive model. This might be particularly relevant in cases with limited data availability [57]. Problem structure is especially important when using the model to guide interventions, such as in precision medicine and genetic engineering. The most exciting advancements will likely occur by combining the general, gradient-based mappings of DL with the symbolic reasoning of HDC into neuro-symbolic AI [151]. Recent work highlighted the potential of such hybrid model [90,152–155] for combining the compositional power of symbolic reasoning with the flexibility and scalability of gradient-based learning.
